# Speech Discrimination Difficulties in High-Functioning Autism Spectrum Disorder Are Likely Independent of Auditory Hypersensitivity

**DOI:** 10.3389/fnhum.2016.00401

**Published:** 2016-08-09

**Authors:** William A. Dunlop, Peter G. Enticott, Ramesh Rajan

**Affiliations:** ^1^Neuroscience Program Biomedicine Discovery Institute, Department of Physiology, Monash UniversityMelbourne, VIC, Australia; ^2^Cognitive Neuroscience Unit, School of Psychology, Deakin UniversityGeelong, VIC, Australia; ^3^Monash Alfred Psychiatry Research Centre, Monash UniversityMelbourne, VIC, Australia; ^4^Ear Sciences Institute of AustraliaPerth, WA, Australia

**Keywords:** autism spectrum disorder, speech-in-noise discrimination, auditory hypersensitivity, auditory attention, auditory behavior questionnaire

## Abstract

Autism Spectrum Disorder (ASD), characterized by impaired communication skills and repetitive behaviors, can also result in differences in sensory perception. Individuals with ASD often perform normally in simple auditory tasks but poorly compared to typically developed (TD) individuals on complex auditory tasks like discriminating speech from complex background noise. A common trait of individuals with ASD is hypersensitivity to auditory stimulation. No studies to our knowledge consider whether hypersensitivity to sounds is related to differences in speech-in-noise discrimination. We provide novel evidence that individuals with high-functioning ASD show poor performance compared to TD individuals in a speech-in-noise discrimination task with an attentionally demanding background noise, but not in a purely energetic noise. Further, we demonstrate in our small sample that speech-hypersensitivity does not appear to predict performance in the speech-in-noise task. The findings support the argument that an attentional deficit, rather than a perceptual deficit, affects the ability of individuals with ASD to discriminate speech from background noise. Finally, we piloted a novel questionnaire that measures difficulty hearing in noisy environments, and sensitivity to non-verbal and verbal sounds. Psychometric analysis using 128 TD participants provided novel evidence for a difference in sensitivity to non-verbal and verbal sounds, and these findings were reinforced by participants with ASD who also completed the questionnaire. The study was limited by a small and high-functioning sample of participants with ASD. Future work could test larger sample sizes and include lower-functioning ASD participants.

## Introduction

Compared to typically developing (TD) people, individuals with Autism Spectrum Disorder (ASD) present a variety of abnormal auditory processing behaviors. Recent changes to the diagnostic criteria for ASD include hyper- and hypo-reactivity to sensory stimuli including sound (DSM-5; American Psychiatric Association, 2013). ASD individuals can show normal or superior performance on some simple psychoacoustic tasks, for example, often excelling at pitch memory and discrimination tasks (Heaton, 2003; O'Riordan and Passetti, 2006; Heaton et al., 2008; Bonnel et al., 2010), and performing as well as age-matched TD controls on simple auditory processing tasks such as distinguishing basic vocalizations like laughter or crying (Jones et al., 2011). However, for tasks that require complex auditory processing such as prosody, speech intonation and visual-auditory integration, participants with ASD perform poorly (see O'Connor, 2012 for a detailed review). In particular, speech processing is often atypical in ASD, especially in experimental conditions that mimic everyday experience, with ASD individuals often reporting difficulty comprehending speech in noisy environments (O'Neill and Jones, 1997).

The deficit in speech-in-noise (SiN) perception appears to depend on the type of background noise: statistically significant, but small differences between TD individuals and those with ASD in SiN perception were found only for background sounds that contained temporal dips and not when the masking noise was temporally flat (Peters et al., 1998; Alcántara et al., 2004). This result suggests that SiN discrimination difficulties in ASD individuals cannot be due to simple energetic (“auditive”) masking of speech sounds (Larsby et al., 2005). The disadvantage when masker noise contains temporal dips may be related to the ability to take advantage of “dip listening” in which listeners hear target speech during brief moments when the noise intensity is lower due to temporal fluctuations (Peters et al., 1998; Alcántara et al., 2004). It is hypothesized (Alcántara et al., 1996) that TD controls are able to use contextual cues and can “piece together” meaning using the limited information gathered in the dips (Miller and Licklider, 1950) whereas ASD listeners are less able to rely on contextual cues (Qian and Lipkin, 2011) and so cannot “piece together” meaning (Alcántara et al., 1996).

In the study by Alcántara et al. (2004), both ASD and TD individuals performed worse in the SiN discrimination task when background noise was a single female speaker with natural temporal dips, compared to when noise contained both temporal (identical to that in the female speaker's utterances) and spectral (four alternating frequency bands) dips (Peters et al., 1998; Alcántara et al., 2004). Because the verbal noise complexity caused greater disruption to SiN discrimination, it is likely that individuals with ASD would have even greater difficulty than TD individuals in more complex background noise such as multi-talker babble noise, which causes greater informational masking in addition to energetic masking (Lutfi, 1990; Bronkhorst, 2000; Brungart, 2001; Brungart et al., 2001; Scott et al., 2004; Rhebergen and Versfeld, 2005; Van Engen and Bradlow, 2007). While not well defined, where speech is used as the target, informational masking makes it harder to segment speech from noise and some segments of the noise are falsely attributed to the target speech (Carhart et al., 1969) likely through interference at lexical, sub-lexical, and/or prosodic levels, and/or phonetic similarity between target sentence and background noise (Van Engen and Bradlow, 2007). Also, multi-talker babble can have an attention-diverting effect (Sperry et al., 1997; Larsby et al., 2005) and given qualitative evidence for individuals with ASD reporting difficulties with auditory attention in noise (O'Neill and Jones, 1997; Birch, 2003; Grandin, 2006; Robison, 2011), such noise may well exacerbate the difficulties of individuals with ASD. We now propose to investigate dysfunctional SiN discrimination in ASD using multi-talker babble noise, which demands greater attention than a single competing talker and speech-weighted noise.

Another auditory processing anomaly prevalent in the ASD population is hypersensitivity to auditory stimulation, which is seen in approximately 40% of ASD individuals and is a stable symptom often observed from very early stages of development (Rimland and Edelson, 1995; Stiegler and Davis, 2010). Indeed, the DSM-5 now includes hyper- or hypo-reactivity to sensory stimulation or unusual interest in sensory aspects of the environment in the diagnostic criteria for ASD (American Psychiatric Association, 2013). Hypersensitivity in a subset of individuals with ASD could not account for a global deficit in SiN discrimination but it may exacerbate poor SiN discrimination in individuals with ASD who have such auditory hypersensitivity. To our knowledge this has not been examined and we propose to also test for this relationship. It is worth noting that evidence for hypersensitivity affecting the performance of individuals with ASD in SiN tasks could support a bottom-up processing deficit model. Reduced tolerance to auditory stimulation could increase perceived noise intensity, thereby increasing effective masking and reducing SiN discriminability. Thus, this testing could also shed light on potential mechanisms affecting auditory processing in ASD individuals.

Finally, we were interested in linking difficulties in SiN perception and auditory hypersensitivity to auditory experiences in daily life. Two questionnaires have been developed for detecting abnormal auditory behaviors in ASD; the Sensory Experience Questionnaire and the Auditory Behaviour Questionnaire (ABQ; Egelhoff and Lane, 2013, SEQ-3.0; Ausderau et al., 2014). While highly detailed, both are specifically designed for a broad range of abnormal behaviors in children and completed by caregivers and this does not allow direct determination of the individual's own experiences. Further, in both, items reflect behaviors that characterize a diagnosis of ASD, so comparisons cannot be easily made with TD and non-ASD clinical groups that contain auditory processing abnormalities. Therefore, for the current study, we designed a questionnaire to measure self-reported auditory behaviors in adults based on validated inventories for specific adult clinical populations that experience abnormal auditory processing (Schow and Nerbonne, 1980; Ventry and Weinstein, 1982; Meijer et al., 2003). The resulting questionnaire, which we call the Auditory Attention and Distress Questionnaire (AADQ), is designed to identify two key auditory processing behaviors, difficulty hearing in a noisy environment and abnormal sensitivity to sounds.

The current study is comprised of two fundamental elements. First, we investigate differences in SiN discrimination between TD participants and those with ASD who are hypersensitive or non-hypersensitive using informational (multi-talker babble) and energetic (speech-weighted noise) maskers. Second, the psychometric properties are assessed for a new questionnaire, designed to measure difficulties hearing speech in typical noisy environments and hypersensitivity to acoustic stimulation in daily life. We hypothesize that (1) SiN discrimination will be poorer in multi-talker babble than in speech-weighted noise; (2) participants with ASD will perform poorly compared to TD controls at speech discrimination in multi-talker babble but not in speech-weighted noise; (3) participants with ASD who are hypersensitive to sound will perform poorly compared to those who are non-hypersensitive in both noise types; (4) the AADQ will divide into a two-component model, measuring Auditory Attentional Difficulties and Auditory Discomfort in everyday life; (5) performance on the SiN task will be reflected in the responses to the AADQ.

## Methods

Procedures were approved by Alfred Health Human Ethics Committee (263/10) and Monash University Human Research Ethics Committee (CF10/2694 – 2010001517) and conformed to the protocols of the Helsinki Declaration and to the guidelines of the National Health and Medical Research Council of Australia for experiments involving humans. Participants were aware that they could withdraw consent without reason at any stage of testing. The current study was divided into two experiments. Experiment 1 involved psychophysical testing of TD participants and those with ASD (see Section Psychophysical Testing). Experiment 2 involved the collection and analysis of pilot data for the AADQ (see Section Design of the AADQ).

### Psychophysical testing

Experiment 1 recruited 28 male and 6 female TD participants aged 19 to 51 (mean = 27, *SD* = 9.84) and 14 males and 2 females with ASD aged 20 to 52 (mean = 34, *SD* = 10.13). These participants were screened for hearing loss then completed a measure of sensitivity to speech and the experimental SiN task. All auditory testing occurred in a quiet room with minimal distractions and conducted between 10 a.m. and 3 p.m.

#### Hearing status exclusion criteria and diagnoses

Hearing status of all participants in Experiment 1 was determined using pure tone audiometry with a Beltone Model 110 Clinical Audiometer and calibrated TDH headphones. Hearing sensitivity was tested at 250, 500, 750, 1000, 1500, 2000, 4000, 6000, and 8000 Hz and thresholds recorded in decibels Hearing Level (dB HL) relative to normal hearing sensitivity (ISO, 1989). The bilateral 4-tone threshold average was calculated using 500, 1000, 2000, and 4000 Hz signals in both ears. All participants included in the current study had <25 dB HL hearing thresholds on the bilateral 4-tone average, and 6000 and 8000 Hz tones, which have been shown to be important for SiN tasks in previous, unpublished laboratory work.

Participants with ASD were diagnosed according to DSM-IV criteria by an experienced psychiatrist, psychologist, or pediatrician, and the diagnostic report was reviewed by the researchers. Two male ASD participants had a diagnosis of High-Functioning Autism and the remaining 14 had a diagnosis of Asperger's Syndrome. The current study was conducted prior to the release of the adult version of the Social Communication Scale (Bölte, 2012), and given time constraints, it was inappropriate to conduct the Autism Diagnostic Interview or the Autism Diagnostic Observation Schedule (Lord et al., 1994, 2002). Instead, the revised Rivto Autism Asperger Diagnostic Scale (RAADS-R; Ritvo et al., 2011) was administered to assess autism traits in the participants with a clinical diagnosis of ASD. Scores ranged from 52 to 190 (mean = 113.77, *SD* = 39.72), with only one participant scoring below the recommended cut-off of 65+ (Ritvo et al., 2011). The participant with a total RAADS-R score of 52 scored moderate to high on all subscales except “Social Anxiety” for which he scored 8 out of a possible 36. We consider that the diagnosis in conjunction with RAADS-R is a sufficient measure to define participant groups as demonstrated in previous studies (Kirkovski et al., 2015, 2016; Palmer et al., 2015).

#### Hypersensitivity to speech sounds

A chart was placed in front of the participant with a hemi-circle drawing numbered 1 to 7 from one end of the hemi-circle to the other. Emoticons were placed at the numbers 1, 4, and 7: a smiley face at 1 to indicate no discomfort, a neutral face at 4 to indicate moderate discomfort and a sad face at 7 to indicate great discomfort. A set of seven three-keyword sentences (see Section Auditory Stimuli for details) were presented binaurally from 60 to 90 dB sound pressure level (SPL) in 5-dB steps. Participants indicated the extent of auditory discomfort for each stimulus by pointing to the number that corresponds to their perceived loudness discomfort level. Loudness Discomfort ratings completed by TD participants were used as baseline sensitivity. Participants with ASD who had loudness discomfort ratings that were similar baseline were considered non-hypersensitive. Those with a score >1.64 standard deviations above baseline (corresponding to 90% confidence intervals) were considered hypersensitive to speech.

#### SiN discrimination

Participants were randomly allocated into one of two groups: multi-talker babble or speech-weighted noise. Each participant was tested with only one type of background noise (see Section Auditory Stimuli). The multi-talker babble condition was completed by 14 TD participants aged 19 to 51 (mean = 28, *SD* = 12) and eight with ASD aged 21 to 50 (mean = 35, *SD* = 9). The speech-weighted noise condition was completed by 12 TD participants aged 19 to 33 (mean = 22, *SD* = 4) and three with ASD aged 34 to 40 (mean = 37, *SD* = 3). There was no overlap between the TD and ASD groups in the speech-weighted noise condition. However, this condition was used to emulate previous research findings and is not the primary experimental goal of the current study.

The general procedures for the SiN task are consistent with those of our previous studies (Burns and Rajan, 2008; Cainer et al., 2008; Rajan and Cainer, 2008; Mann et al., 2013). In each block, continuous noise was turned on 5 s before the first three-keyword sentence was presented (see Section Auditory Stimuli), and participants verbally repeated the sentence or indicated that they were unable to do so. A correct response required all three keywords to be identified in the correct order. There was no time limit for a response and feedback was not given. Once the response was recorded by the experimenter on the in-house program, a new sentence was selected at random and presented after a 1.5-s delay. To avoid audibility issues, all sentences were presented at 70 dB SPL. Five blocks, each with 20 unique sentences, were each allocated a specific SNR. For both noise conditions, in the first block, noise was presented 1 dB lower than the sentence (SNR = 1) to ensure that the first block was not too difficult. In the multi-talker babble condition, subsequent blocks were randomized such that noise was presented at SNRs of 3, −1, −3, or −5. In the speech-weighted noise condition, subsequent blocks were presented in random order at SNRs of −1, −3, −5, or −7.

#### Auditory stimuli

Target sentences were pre-recorded in a female voice with an Australian accent in a neutral tone, using standardized sentences (BKB; Bench et al., 1979). The full BKB list contains 192 sentences, four to six words long, three of which are keywords. In a separate pilot study, psychometric functions were determined for all 192 BKB sentences delivered at a fixed-intensity at different levels of speech weighted noise for 15 young, normal-hearing participants and the speech reception threshold for each sentence was determined by the SNR at which 50% of participants could correctly reproduce the sentence (SNR-50; Plomp and Mimpen, 1979; Nilsson et al., 1994; van Wijngaarden et al., 2002; Killion et al., 2004). Of the 192 BKB sentences, 100 were identified to have similar speech reception thresholds. These were divided into five lists of twenty and each list was assigned to a specific SNR condition. A one-way ANOVA confirmed no difference in speech reception thresholds between lists, *F*_(4, 95)_ = 0.22, *p* = 0.90. Due to the limited number of standardized sentences available with similar psychometric properties, the same sentences were used for both speech-weighted noise and multi-talker babble conditions. Therefore, participants could not complete both noise conditions because they would have prior knowledge of the BKB sentences used. BKB sentences that were used for measuring speech-hypersensitivity were selected from the remaining 92 BKB sentences.

Two types of noises were used for SiN discrimination tasks, multi-talker babble and speech weighted noise (Rajan and Cainer, 2008). Multi-talker babble was generated by recording four people reading nonsense text. The recording was doubled-over and temporally offset to create the impression of eight simultaneous voices to model what has been shown to be the most effective babble masker, at least for phoneme detection. As demonstrated by Simpson and Cooke (2005), for phoneme detection, increasing the number of competing speakers in the background babble noise from 1 to 8 increases phoneme identification difficulty, but beyond 8, difficulty decreases until at 512 speakers. With a large number of speakers, babble noise is only as effective as speech-weighted noise, a purely energetic masker of speech sounds (Simpson and Cooke, 2005). Speech-weighted noise had a shaped spectrum equal to the long-term average spectrum of the target sentences, as recorded from a Madsen audiometer. Multi-talker babble and speech-weighted noise were adjusted such that they had equal root mean square values.

Speech, multi-talker babble and speech-weighted noise were stored as. WAV files on a Dell Inspiron computer, which was linked to a Creative Sound Blaster Audigy external sound card. Signals generated by the soundcard were presented to participants binaurally through Sennheiser HD535 headphones. The SiN discrimination task was run using an in-house program (designed by Dr Chris James formerly of the Bionic Ear Institute, University of Melbourne), to control delivery of test sentences and background noise, to vary noise level, and to store and display results. For the loudness discomfort level assessments, stimuli were played manually from.WAV files using the same program. For calibration, the headphones were coupled to a Brüel and Kjær Artificial Ear Type 4152 containing a Brüel and Kjær 1 Condenser Microphone Type 4145. The microphone output was connected to a Brüel and Kjær Precision Sound Level Meter Type 2203 on which SPL was directly read off (A-weighted scale on “Slow” time setting), or to a Brüel and Kjær, 2260 Investigator system. Sentence level was calibrated using a reference 1 kHz signal, with average root mean square level set to the same value as for the sentences, and also stored on the computer as.WAV. The noise masker was calibrated by playing the noise through the headphones and again, using the “Slow” time settings to measure output level.

### Design of the AADQ

Experiment 2 recruited 128 TD participants aged 18 to 67 (mean = 36, *SD* = 12.62) to build pilot psychometric data on the AADQ. These participants completed the questionnaire only and were not screened for hearing loss. Of the 16 participants with ASD from Experiment 1, 14 completed the AADQ.

The AADQ was designed to identify two key auditory processing behaviors: difficulty hearing in a noisy environment and abnormal sensitivity to sounds. TD participants completed the AADQ online or on paper and all participants with ASD completed the questionnaire on paper during the single testing session in which they participated. We acknowledge that the sample size is not adequate to provide substantial normative data. However, for the interests of this study, we will interpret the pilot data to consider different auditory behaviors. We are confident that each item holds some validity because the questionnaire was based on validated inventories for specific adult clinical populations that experience abnormal auditory processing; the Hearing Handicap and Denver Scales (Schow and Nerbonne, 1980), the Hearing Handicap Inventory for the Elderly (Ventry and Weinstein, 1982), the Amsterdam Inventory for Auditory Disability and Handicap (Meijer et al., 2003) and an unpublished inventory developed at The University of Auckland for hearing aid users. From these questionnaires, we developed a 33-item questionnaire with statements designed to reflect behaviors in response to environmental sounds, including both annoyance caused by sounds (e.g., “I find traffic noises to be uncomfortably loud”) and impaired ability to concentrate on verbal stimuli in a noisy environment (e.g., “I have trouble understanding a waiter/waitress in a quiet restaurant”). A complete list of items is attached in Appendix A. Participants responded on a seven-point Likert scale with 1 indicating strong disagreement and 7 indicating strong agreement with the statement. Items 8, 17, and 31 have negative valence (e.g., “I can understand conversations even when several people are talking”) and so participants' responses were reversed. Therefore, high-scoring individuals are assumed to be either less able to concentrate in a noisy environment, sensitive to environmental sounds or both.

## Results

Hypersensitive and non-hypersensitive ASD participants were determined based on Loudness Discomfort ratings. SiN discrimination performance was compared between TD, hypersensitive and non-hypersensitive ASD participants using multi-talker babble and speech-weighted noise. Then, performance was related to responses on the AADQ. Except where specified, all analyses were run on IBM SPSS Statistics 22.

### Hypersensitivity

**Figure 1:**
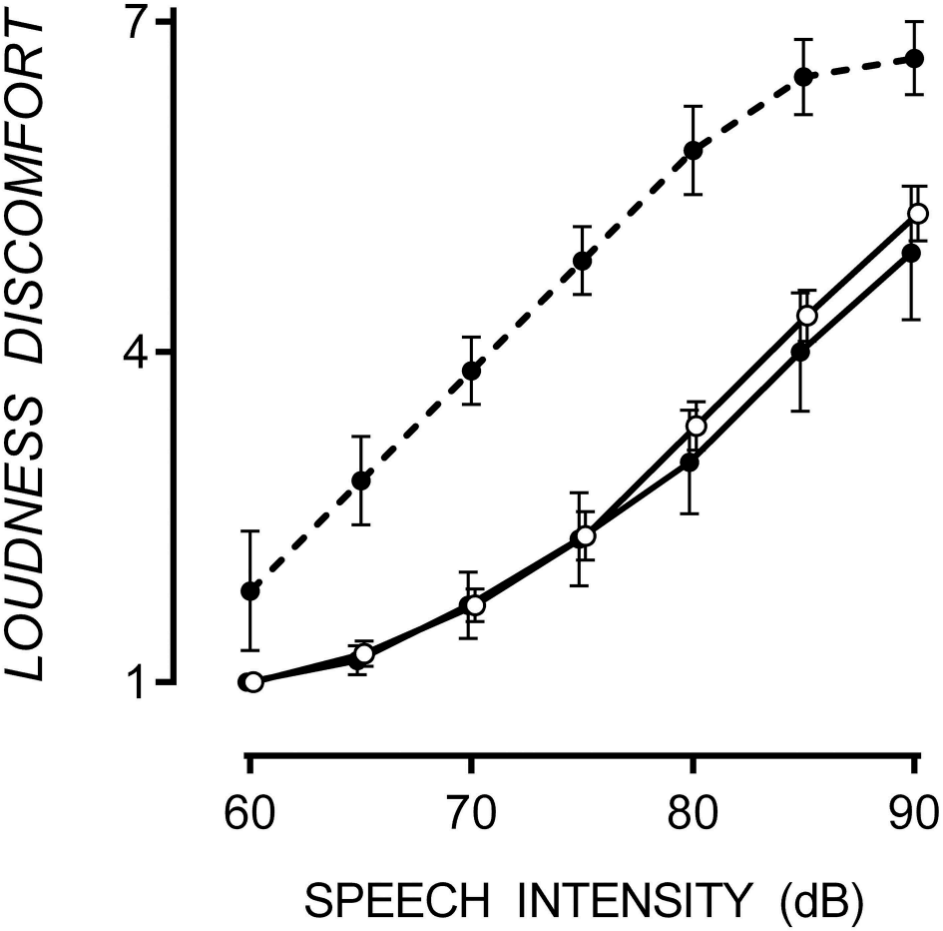
**Mean Loudness Discomfort ratings for speech with one standard error**. Hypersensitive ASD participants (filled circle, dashed line) rated Loudness Discomfort to speech higher than TD (empty circles) and non-hypersensitive ASD participants (filled circles, solid line), *p* < 0.001. Data points are offset for visibility.

ASD participants were classified into hypersensitive and non-hypersensitive groups based on whether they rated Loudness Discomfort beyond the 90% confidence intervals of TD mean. Responses to Loudness Discomfort were compared to confirm between-group differences. ASD participants were considered hypersensitive to speech if they rated any signal intensity >1.64 standard deviations above the mean collected from TD participants in that condition. Six of the16 participants with ASD were hypersensitive to speech, similar to the 40% prevalence in the ASD population (Rimland and Edelson, 1995; Stiegler and Davis, 2010). Loudness discomfort ratings for TD participants and those with ASD (both hypersensitive and non-hypersensitive are shown in Figure 1.

A 3 × 7 [(TD, hypersensitive and non-hypersensitive ASD) × (60, 65, 70, 75, 80, 85, and 90 dB)] two-way mixed ANOVA supported a difference in Loudness Discomfort ratings for speech between groups, *F*_(12, 72)_ = 2.04, *p* = 0.033. Speech-hypersensitive ASD participants (6/16) rated Loudness Discomfort to speech 1.87 (*SE* = 0.38) and 2.03 (*SE* = 0.43) points higher than TD and non-hypersensitive ASD participants respectively, both significant at the *p* < 0.001 level. There was no difference in Loudness Discomfort between TD and non-hypersensitive ASD participants, *p* = 0.862.

### SiN discrimination

**Figure 2:**
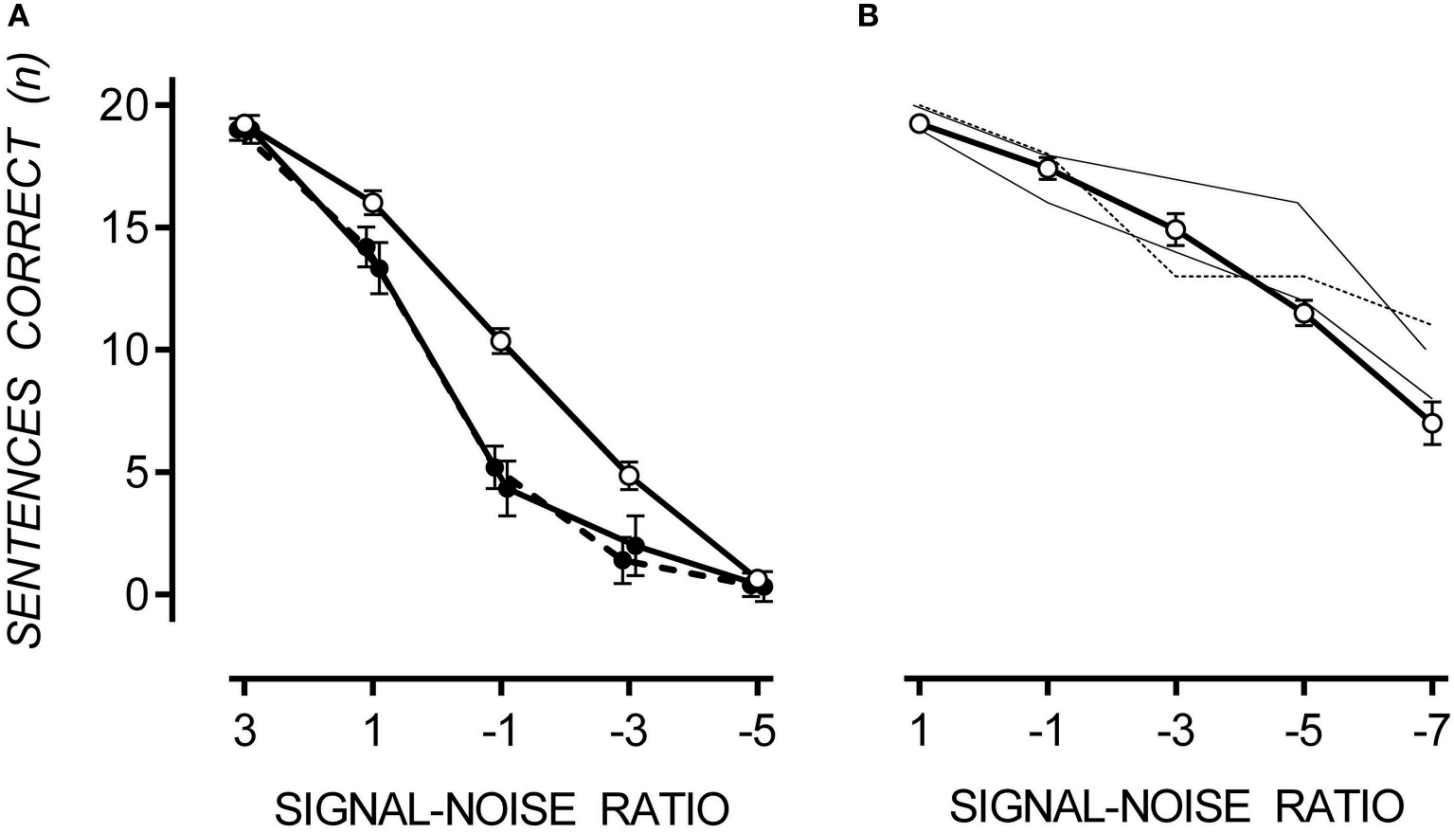
**Mean performance on the SiN discrimination task with one standard error**. In multi-talker babble **(A)**, participants with ASD (filled circles), both hypersensitive (dashed line) and non-hypersensitive (solid line), performed poorly at SiN discrimination compared to TD participants (empty circles), *p* = 0.022. Data points are offset for visibility. In speech-weighted noise **(B)**, participants with ASD (thin dashed line for hypersensitive and thin solid line for non-hypersensitive) performed no different to TD participants (open circles), except for one participant at a SNR of −5 who scored 2.68 standard deviations above the TD mean.

In both noise conditions, sentence recall decreased as SNR decreased. A floor effect occurred in multi-talker babble at a SNR of −5, at which point sentence recall was equally poor for TD and ASD participants. In multi-talker babble, only 27% of participants could correctly recall one or more sentences at a SNR of −5. However, in speech-weighted noise, all participants could correctly recall approximately 50% of sentences at a SNR of −7. Therefore, it appears that SiN discrimination was more difficult in multi-talker babble. However, a direct comparison between noise conditions cannot be made due to the between-groups design. Mean and standard error of correctly recalled sentences for TD, hypersensitive and non-hypersensitive ASD participants are presented in Figure 2.

#### Multi-talker babble

A 3 × 5 [(TD, hypersensitive and non-hypersensitive ASD) × (SNR = 3, 1, −1, −3, and −5)] two-way mixed ANOVA was conducted to measure the difference in sentence recall in multi-talker babble between TD participants and those with ASD, both hypersensitive and non-hypersensitive. Decreasing SNR reduced the number of sentences correctly reproduced from 19.07 (*SE* = 0.26) at a SNR of 3 to 0.46 (*SE* = 0.27) at a SNR of −5, *F*_(4, 16)_ = 692.28, *p* < 0.001. There was a factorial difference between groups, *F*_(8, 34)_ = 2.67, *p* = 0.022, observed power was 0.86, and Tukey HSD revealed that TD participants correctly reproduced 2.41 (*SE* = 0.70) and 2.17 (*SE* = 0.57) more sentences than participants with ASD who were hypersensitive, Cohen's *d* = 1.99, *p* = 0.007, and non-hypersensitive, Cohen's *d* = 2.02, *p* = 0.003 respectively. There was no difference between hypersensitive and non-hypersensitive ASD participants, *p* = 0.952.

Boltzmann sigmoidal functions were fitted to the data using GraphPad PRISM 6 to estimate the psychometric functions of SiN with multi-talker babble for TD participants and those with ASD, both hypersensitive and non-hypersensitive. The top and bottom of the functions were constrained to 20 and 0 respectively. Data are presented in Table 1. Estimates for goodness of fit were very strong for each group. The slope of the function for TD participants was less steep than that of participants with ASD, regardless of hypersensitivity, *F*_(2, 98)_ = 4.44, *p* = 0.014. The midpoint was at a lower SNR for TD participants compared to those with ASD, regardless of hypersensitivity, *F*_(2, 98)_ = 20.02, *p* < 0.001.

**Table 1:** **Goodness of fit, slope and midpoint values for Boltzmann sigmoidal functions for TD participants and those with ASD, both hypersensitive and non-hypersensitive**.

	***df***	***R*^2^**	**Slope**	***SE*_slope_**	**Midpoint**	***SE*_midpoint_**
TD	66	0.95	2.08	0.45	−1.32	0.29
Hypersensitive	11	0.93	1.02	0.38	0.45	0.43
Non-hypersensitive	21	0.97	1.03	0.18	0.15	0.021

#### Speech-weighted noise

Due to small sample of participants with ASD completing the SiN task in speech-weighted noise, individual performance was compared with data collected from TD participants. A difference in performance was considered significant if it was >1.64 standard deviations from the TD mean. Participants with ASD performed no differently compared to the TD mean except for one participant (non-hypersensitive ASD) who scored 2.68 standard deviations above the TD mean at a SNR of −5.

#### Pilot data on the AADQ collected from TD participants only

**Figure 3:**
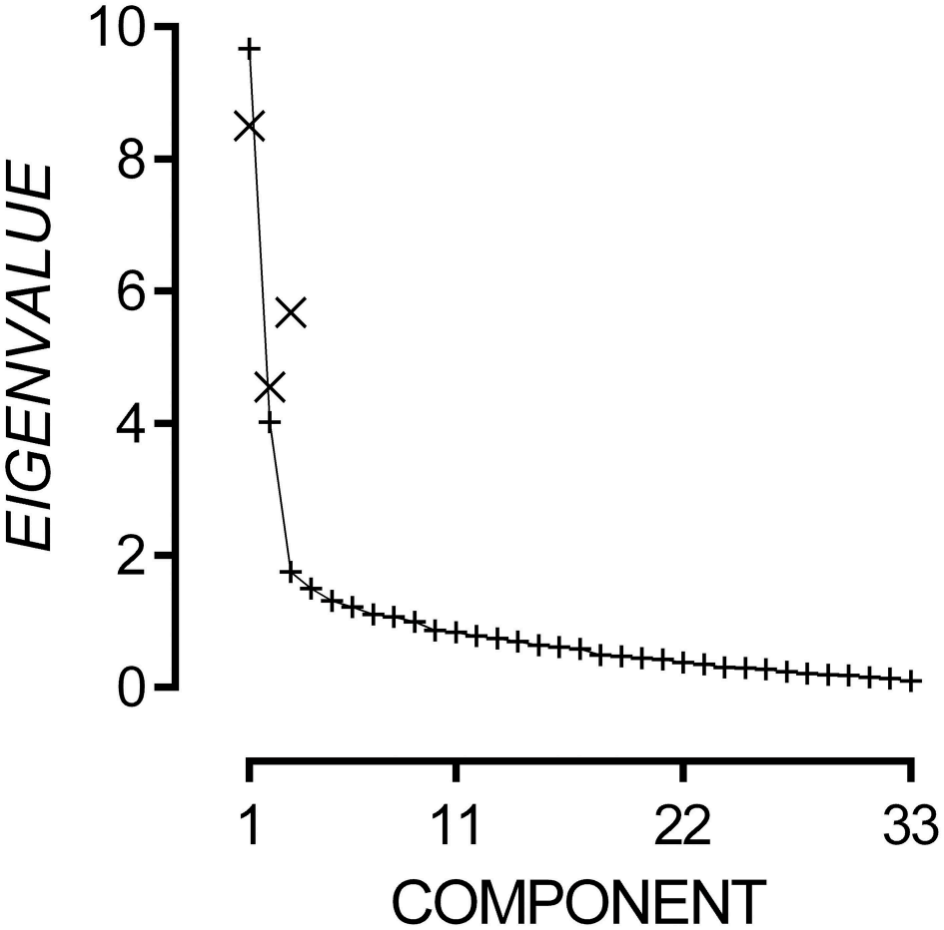
**Plot of non-rotated eigenvalues (+) for each possible component and rotated eigenvalues (×) for Components 1, 2, and 3**.

The 33-item questionnaire data had strong internal consistency, Cronbach's α = 0.90, and good sampling adequacy, KMO = 0.83, χ^2(528)^ = 2174.37, *p* < 0.001. Multicollinearity was not detected using a Variance Inflation Factor cut-off of 3. Due to significant inter-item correlations, Principal Components Analysis was conducted with oblique rotation. Inspection of the scree plot presented in Figure 3 reveals that eigenvalues flatten after the third component and can add only small incremental amounts to the variance explained. Hence, we applied a three-component model to the questionnaire outcomes.

The three-component model explained 46.76% of variance. Components 1, 2, and 3 had rotated eigenvalues of 8.51, 4.55, and 5.68 respectively. The pattern matrix for the three-component model is presented in the Appendix A. Items 5, 7, 8, and 17 did not load onto any component and were excluded. Fourteen items loaded onto Component 1, and all appeared to measure difficulties attending to speech in noisy environments. Therefore, Component 1 will hereto be referred as the Audio-Attentional Difficulty subscale. Eight items loaded onto Component 2, and all appeared to measure discomfort to non-verbal environmental sounds. Therefore, Component 2 will hereto be referred as the Auditory Discomfort (Non-Verbal) subscale. Five items loaded onto Component 3, and all appeared to measure discomfort to verbal sounds. Therefore, Component 3 will hereto be referred as the Auditory Discomfort (Verbal) subscale. Subscale scores were generated by summing the response to each item that loaded onto it. Audio-Attentional Difficulty had a possible range of 14–98, Auditory Discomfort (Non-Verbal) had a possible range of 8–56, and Auditory Discomfort (Verbal) had a possible range of 5–35.

#### Applying data from participants with ASD to the normative pilot data

With TD and ASD scores combined, skewness was greater than twice the standard error of the skew on all subscales, so a logarithmic transformation was used to successfully normalize each distribution. Pearson's *r* was used to estimate the relationship between the three subscales. Auditory Discomfort (Non-Verbal) had a moderate positive relationship with Audio-Attentional Difficulty, *r*_(142)_ = 0.43, *p* < 0.001 and with Auditory Discomfort (Verbal), *r*_(142)_ = 0.50, *p* < 0.001. There was a strong positive relationship between Audio-Attentional Difficulty and Auditory Discomfort (Verbal), *r*_(142)_ = 0.69, *p* < 0.001.

One-way ANOVAs were conducted to measure the difference between TD participants and those with ASD, both hypersensitive and non-hypersensitive, on all three subscales. There were significant group differences on Audio-Attentional Difficulty [*F*_(2, 139)_ = 21.14, *p* < 0.001], Auditory Discomfort (Non-Verbal) [*F*_(2, 139)_ = 9.05, *p* < 0.001] and Auditory Discomfort (Verbal) [*F*_(2, 139)_ = 14.61, *p* < 0.001]. *Post-hoc* analysis was conducted using Tukey HSD Homogeneous Subsets due to unequal group sample sizes (Smith, 1971).

On the Audio-Attentional Difficulty scale, hypersensitive and non-hypersensitive participants with ASD scored no differently from each other (*p* = 0.065), and both groups scored higher than TD participants, *p* < 0.001). On the Auditory Discomfort (Non-verbal) scale, TD participants scored the same as non-hypersensitive participants with ASD (*p* = 0.442) and both groups scored lower than hypersensitive participants with ASD (*p* < 0.05). Similarly, on the Auditory Discomfort (Verbal) scale, TD participants scored the same as non-hypersensitive participants with ASD (*p* = 0.118) and both scored lower than hypersensitive participants with ASD (*p* < 0.001).

## Discussion

The current study was comprised of two primary investigations of auditory processing behaviors in ASD compared to TD participants: (1) Psychophysical testing of SiN discrimination in different noise backgrounds, in relation to hypersensitivity, and (2) Questionnaire-based testing of loudness related measures in daily life, given preliminary evaluation with psychometric analysis. The first provides novel evidence for a top-down SiN processing deficit in ASD compared to TD individuals. The second provides a useful tool for distinguishing between Audio-Attentional Difficulty and two subtypes of Auditory Discomfort, Non-Verbal and Verbal, the distinction of which is novel to the literature.

The diagnosis of ASD was confirmed with participants' clinicians by the researchers by reviewing the diagnostic report. To further support the validity of these diagnoses, participants with ASD completed the RAADS-R. One participant scored 52, 13 points below the cut-off of 65+ (Ritvo et al., 2011). However, this participant had moderate to high scores on all subscales except for social anxiety and performed no differently to the other participants with ASD. It is of note that despite heterogeneity of speech sensitivity within the ASD sample, performance on the SiN task was homogenous between subgroups of participants with ASD.

### Loudness tolerance and SiN discrimination

Individuals with ASD performed poorly compared to age-matched TD controls in speech discrimination when speech was presented in multi-talker babble, and there was no evidence that speech-hypersensitivity predicted performance. Similarly, in speech-weighted noise, speech-hypersensitivity did not predict performance in SiN discrimination. Therefore, the perceived energetic intensity of the stimuli does not affect the ability to discriminate speech from noise in our sample. The findings are consistent with Alcántara et al. (2004), who demonstrated that TD participants outperformed those with ASD in SiN discrimination when noise was temporally shaped, but not when it was temporally flat. One difference between the current findings and those of Alcántara et al. (2004) is that our data suggest that both TD and ASD participants perform poorly in temporally shaped noise compared to temporally flat noise. The between-groups design limits the conclusion that SiN discrimination is more difficult in temporally-shaped noise; however, it is consistent with Simpson and Cooke (2005), which demonstrates that phoneme discrimination is more difficult in 8-speaker babble compared to single competing speaker and speech-weighted noise conditions. Our findings contribute further by providing supportive evidence that SiN discrimination deficits in ASD appear to be independent of hypersensitivity to speech.

The current research indicates that the disadvantage in participants with ASD is not due to a generalized difficulty in extracting a signal from noise *per se*: from the small sample tested, there was no evidence that speech-weighted noise reduced the ability to correctly reproduce target sentences for hypersensitive and non-hypersensitive ASD participants more than for TD participants, which is consistent with the findings by (Alcántara et al., 2004). Further, even in multi-talker babble, all three groups of subjects did equally well at the favorable SNR of 3 dB. These findings are consistent with the literature (O'Connor and Kirk, 2008; Rizzolatti and Fabbri-Destro, 2008; Barbalat et al., 2014; Bodner et al., 2014), which argues that it is unlikely that SiN deficits are the result of difficulty comprehending speech, deficient verbal short-term memory or cognitive deficits, which have been reported in individuals with ASD. We are therefore confident that differences in cognitive and verbal intelligence are unlikely to be solely responsible for the SiN processing deficits in our ASD sample, despite no formal measurements being made.

Previous studies that used broadband or speech-weighted noise to mask signals indicate that individuals with ASD can extract basic tonal (Bonnel et al., 2010) and speech (Alcántara et al., 2004; Groen et al., 2009) information from masked noise if it is temporally flat. Therefore, it is unlikely that poor SiN discrimination results from abnormal processing in the auditory periphery (i.e., cochlear masking mechanisms suppressing the response to noise). Instead, we postulate that attention deficits prevent the discrimination of target speech from informational masking noise. Discriminating perceptually similar signals requires the listener to use vocal cues like pitch and intonation (Cainer et al., 2008), and contextual cues like semantic structure and anticipation (Miller and Licklider, 1950; Groen et al., 2009) within the target speech. Therefore, the disadvantage among participants with ASD in multi-talker babble appears to be a difficulty in extracting sentences in the presence of temporally complex noise that reduces speech discrimination abilities for both TD and ASD individuals, but more so in individuals with ASD.

In summary, our results show that SiN discrimination difficulty in individuals with high functioning ASD is unlikely to be the result of abnormal sensitivity to sound, an inability to extract signal from noise due to energetic masking, nor verbal cognitive impairments. Two possible explanations include an attention deficit that inhibits ASD individuals' ability to attend to target speech over complex noise, and poor use of speech cues that prevents ASD participants from following target speech.

A factor analytic study of the auditory abilities underlying the recognition of familiar sounds (like the simple sentences used in this study), especially for recognition of auditory stimuli under conditions of limited or distorted information, suggested that this ability likely depended on three factors: (a) rapid access to the lexicon, (b) more effective use of stimulus knowledge in a problem-solving strategy to fill in missing gaps (which we interpret to mean better use of contextual cues in the sentences we used), and (c) more effective active and dynamic focusing of attention on the most informative spectro-temporal locations within a familiar sound (Kidd et al., 2007). The sentences we used are simple and the ASD participants performed equally to the TD participants for the same sentences in a background of speech-weighted noise, and at an optimal SNR in a background of babble noise. We argue that these facts make it unlikely that either lexical access constraints in ASD participants or improvements in TD participants, or differences in the ability to use contextual cues, was a contributor to the poorer performance of ASD participants at less favorable SNRs in babble noise. This leaves the possibility that the ASD participants made poorer use of active, dynamic attentional focusing in the babble noise to extract the requisite information to identify the sentences. It must be noted that this information could also be contextual information to allow sentence reconstruction and recall based on stimulus knowledge, and it could be argued that this fits with the second proposal of Kidd et al. (2007) against which we have just argued. However, as our proposal is that this poorer use of contextual information arises from the deficiency in the third factor, we argue that this third factor—poorer active dynamic attention focusing in babble noise—appears to be the most reasonable explanation for the poorer performance of ASD participants at less favorable SNRs in babble noise.

The basic principle of auditory masking is that the energetic intensity of noise competes with that of the to-be-detected signal. The more similar the noise is to the signal, the harder it is to discriminate between the two (Moore, 2007). The current study demonstrates that in both TD and ASD groups, multi-talker babble, containing speech sounds, was a more effective masker of target speech than was temporally flat speech-weighted noise, perceptually different from speech. These findings are consistent with the distinction between informational and energetic masker noise (Brungart, 2001; Brungart et al., 2001), and suggest an attention deficit in ASD could be related to poor SiN discrimination.

Posner and Petersen (1990) proposed three subtypes of attention, namely alerting, orienting and executive attention. Alerting attention, which involves state arousal (Petersen and Posner, 2012), is unlikely to be related to poor performance in ASD because there were no group differences in speech-weighted noise or in multi-talker babble when the SNR was most favorable. Orienting attention, based on stimulus location (Petersen and Posner, 2012), pertains to visual processing and is therefore unlikely associated with the differences observed between TD and ASD performance in SiN tasks. Executive attention, however, allows suppression of competing sensory input streams (within and between modalities) in favor of one target stream (Petersen and Posner, 2012), and is likely important in SiN discrimination. In discrimination tasks, both visual and auditory, non-target stimuli cause little load on the attention system, but multiple target stimuli cause great disruption (Duncan, 1980). Multi-talker babble, which contains speech sounds similar to the target speech, would place greater strains on cognitive load and thereby decrease speech processing efficiency. Those with ASD, who often exhibit deficits in executive attention (Ozonoff et al., 1991; Samyn et al., 2014; Troyb et al., 2014; Uddin et al., 2014) would be disadvantaged compared to TD individuals when attention-demanding noise is used.

The noise containing temporal dips in the current study effectively used eight competing talkers, which is more difficult than a single competing speaker for phoneme identification, a vital element of speech comprehension (Simpson and Cooke, 2005). It is possible that increasing the number of speakers would cause vocalizations to overlap, reducing the number of temporal dips available in which a listener can catch glimpses of the target speech (Miller and Licklider, 1950; Simpson and Cooke, 2005). Indeed, when the number of simultaneous speakers in multi-talker babble is sufficiently high, the masking effect of noise is equivalent to that of temporally flat speech-weighted noise (Simpson and Cooke, 2005). Future research that compares TD and ASD psychometric functions of speech discrimination with increasing numbers of competing speakers would provide useful insight into dip listening strategies in ASD.

Assuming the difference in SiN discrimination between TD and ASD participants is due to a disadvantage in dip listening, the evidence is consistent with the “Lookup Table” and “Interpolation” learning styles theory proposed by Qian and Lipkin (2011). The theory proposes that ASD individuals store specific examples of sensory input and do not interpolate to create generalized rules to extract abstract patterns. TD participants are able to use glimpses of target speech detected in temporal dips and interpolate the available information based on typical semantic structures and social expectations to derive a possible meaning. Individuals with ASD, favoring a lookup table learning style, cannot derive meaning from glimpses of target speech using existing examples and are less able to interpolate meaning. Follow up testing in which target sentences make no social or semantic sense, similar to experiments by Boothroyd and Nittrouer (1988) could be useful in testing whether TD participants are using social and semantic cues to improve SiN discrimination.

### Loudness discomfort and difficulties in daily activities

We designed a 33-item questionnaire that was found to measure three auditory processing behaviors: Audio-Attentional Difficulty, Auditory Discomfort (Non-Verbal) and Auditory Discomfort (Verbal). Despite small sample sizes, the questionnaire has good psychometric properties, showing strong internal consistency and reliability. Further, group differences in each subscale between TD participants and those with ASD were consistent with Loudness Discomfort ratings and performance on the SiN. Audio-Attentional Difficulty scores were significantly higher for participants with ASD than for TD participants, consistent with performance in the signal discrimination task in multi-talker babble, and Auditory Discomfort (Verbal) and (Non-Verbal) were significantly higher for hypersensitive ASD participants compared to non-hypersensitive ASD and TD participants. While further testing with larger samples is needed to establish the psychometric properties of the AADQ, it appears to be a sensitive tool for distinguishing different auditory behaviors that is relevant to auditory processing research.

Interestingly, despite expecting hypersensitivity to form one uniform component, two distinct Auditory Discomfort subscales were identified in the TD sample. The novel difference between sensitivity to non-verbal and verbal signals suggests that loudness tolerance is at least somewhat dependent on physical properties of the signal other than intensity. A loud non-speech sound can be perceived as irritating but a loud speech sound could be perceived as yelling. An alternative explanation to differences in hypersensitivity in ASD is that Loudness Discomfort is dependent on internal processes such as anxiety and sound source familiarity (Stiegler and Davis, 2010). It is possible that general noise and speech sounds cause different levels of emotional distress, thereby affecting the perceived intensity. By contrast, ASD individuals have aberrant brainstem encoding of speech sounds compared to TD controls (Russo et al., 2009) but not of non-verbal sounds. It is therefore possible that perceived loudness is dependent on early-level processing of non-verbal and verbal sounds. However, the psychometric distinction was also present in the TD sample, indicating that there is a perceived difference in the loudness discomfort caused by auditory stimulation that is independent of intensity, even in non-clinical populations. Systematically comparing Loudness Discomfort ratings for non-verbal sounds (i.e., pure tones and speech-weighted noise) and verbal sounds (i.e., speech and multi-talker babble) between TD and ASD individuals is needed to clarify the processes that underpin auditory hypersensitivity. Understanding the processes that determine Loudness Discomfort in ASD could have implications for why many with an ASD find noisy social situations to be overwhelming (Kanner, 1968; Grandin, 2006; Robison, 2011).

## Conclusions

Despite normal SiN discrimination in speech-weighted noise, individuals with ASD are disadvantaged compared to TD individuals in multi-talker babble. Further, we found no evidence that speech-hypersensitivity was related to performance in the speech-in-noise task. Within the ASD group, hypersensitivity appeared, at least in part, to be dependent on physical properties of the signal other than intensity. However, missing Loudness Discomfort data for multi-talker babble make interpretations difficult. The signal-dependent Loudness Discomfort ratings are consistent with the psychometric properties of the AADQ, which provides evidence that Auditory Discomfort can be categorized into Non-Verbal and Verbal subscales. The current study promotes the need for rigorous testing to determine the processes involved in early auditory perception that determine Loudness Discomfort in ASD. Future studies could address some of the current limitations by recruiting lower-functioning participants with ASD and correlating SiN performance with ASD severity. Further, the AADQ should be tested on a broader population, including non-ASD clinical groups.

## Author contributions

RR and PE were responsible for project conception and study design. PE recruited participants. RR executed the experiments and collected data. WD analyzed the data and wrote the manuscript. All authors contributed to editing the manuscript and have approved of the final submission.

## Funding

The project was supported by internal lab funds. PE is supported by a Career Development Fellowship from the National Health and Medical Research Council (NHMRC), Australia (Grant Number: GNT1052073).

### Conflict of interest statement

The authors declare that the research was conducted in the absence of any commercial or financial relationships that could be construed as a potential conflict of interest.

## Appendix A

Retained items of the Auditory Attention and Discomfort Questionnaire (AADQ) are presented with oblique-rotated factor loadings based on normative data from 128 typically developing participants.

**Table:**

**Item number and statement**		**Audio-attentional difficulty**	**Auditory discomfort (Non-verbal)**	**Auditory discomfort (Verbal)**
1	I find the sound of doorbells annoyingly loud.		0.65	
2	I find the sound of a telephone ringing to be uncomfortably loud.		0.68	
3	I find restaurants and cafes to be uncomfortably loud.			0.77
4	I have arguments with my family or friends because I think they talk too loudly.		0.50	
6	I find supermarkets to be uncomfortably loud.			0.52
9	The sounds of building work are painfully loud.		0.75	
10	Traffic noises are uncomfortably loud.		0.75	
11	The sound of screeching tires is uncomfortably loud.		0.71	
12	When I am in a theater watching a movie or play, I find it uncomfortably loud when people around me are whispering and rustling packets.		0.49	
13	I have trouble understanding others when an air conditioner or fan is on.			0.48
14	Unexpected sounds, like a smoke detector or alarm bell, are uncomfortable.		0.63	
15	I avoid social gatherings (like parties) because I find the noise levels annoying.			0.45
16	I find parties are too loud to be able to concentrate to have a conversation.			0.66
18	I have difficulty hearing a conversation when I'm with one of my family at home.	0.61		
19	I have difficulty following a conversation on the phone or mobile when I'm at home.	0.56		
20	When I am having a quiet conversation with a friend, I have difficulty understanding them.	0.82		
21	When I'm seeing my doctor in his/her rooms, it is hard to follow the conversation.	0.67		
22	I have trouble understanding dialogue in a movie or at the theater.	0.59		
23	When I am talking with someone across a large empty room, I have difficulty understanding what they say.	0.72		
24	I miss a lot of information when I'm listening to a lecture or a public talk.	0.72		
25	When a speaker is addressing a small group, and everyone is listening quietly, I have to strain to hear.	0.71		
26	I have to ask people to repeat themselves in one-on-one conversations in a quiet room.	0.90		
27	I have trouble understanding a waiter/waitress in a quiet restaurant.	0.75		
28	When I am in a small office, talking or answering questions, I have difficulty following the conversation.	0.82		
29	When I am having dinner with several other people, I have difficulty following the conversation because I find it hard to identify who is speaking.	0.56		
30	I have difficulty communicating with others when we are in a crowd.	0.52		
31	When I am in a crowded supermarket talking with the cashier, I can follow the conversation.			0.55
32	I have difficulty understanding a shop assistant in a crowded shop.			0.54
33	In social situations I often feel left out because people think I have difficulty following the conversations.	0.60		

*Items that did not load onto a factor and individual factor loadings < 30 are not shown*.
